# IL6 Inhibits HBV Transcription by Targeting the Epigenetic Control of the Nuclear cccDNA Minichromosome

**DOI:** 10.1371/journal.pone.0142599

**Published:** 2015-11-18

**Authors:** Gianna Aurora Palumbo, Cecilia Scisciani, Natalia Pediconi, Leonardo Lupacchini, Dulce Alfalate, Francesca Guerrieri, Ludovica Calvo, Debora Salerno, Silvia Di Cocco, Massimo Levrero, Laura Belloni

**Affiliations:** 1 Dept of Internal Medicine–DMISM, Sapienza University, Rome, Italy; 2 Dept of Molecular Medicine, Sapienza University, Rome, Italy; 3 INSERM U1052, Cancer Research Center of Lyon (CRCL), Lyon, France; 4 Center for Life NanoSciences (CLNS), IIT-Sapienza, Rome, Italy; Yonsei University, REPUBLIC OF KOREA

## Abstract

The HBV covalently closed circular DNA (cccDNA) is organized as a mini-chromosome in the nuclei of infected hepatocytes by histone and non-histone proteins. Transcription from the cccDNA of the RNA replicative intermediate termed pre-genome (pgRNA), is the critical step for genome amplification and ultimately determines the rate of HBV replication. Multiple evidences suggest that cccDNA epigenetic modifications, such as histone modifications and DNA methylation, participate in regulating the transcriptional activity of the HBV cccDNA. Inflammatory cytokines (TNFα, LTβ) and the pleiotropic cytokine interleukin-6 (IL6) inhibit hepatitis B virus (HBV) replication and transcription. Here we show, in HepG2 cells transfected with linear HBV monomers and HBV-infected NTCP-HepG2 cells, that IL6 treatment leads to a reduction of cccDNA-bound histone acetylation paralleled by a rapid decrease in 3.5kb/pgRNA and subgenomic HBV RNAs transcription without affecting cccDNA chromatinization or cccDNA levels. IL6 repressive effect on HBV replication is mediated by a loss of HNF1α and HNF4α binding to the cccDNA and a redistribution of STAT3 binding from the cccDNA to IL6 cellular target genes.

## Introduction

IL-6 is a pleiotropic cytokine that mediates inflammation and regulates cell growth, differentiation and survival [1]. IL6 acts via receptor complexes containing at least one subunit of the signal-transducing protein gp130. Hetero-dimerization of IL6/gp130 leads to the activation of the intra-cytoplasmic JAK tyrosine kinases (Janus family tyrosine kinases) that phosphorylate and activate STAT3, which in turn dimerize and translocate to the nucleus to activate gene expression [1]. This type of signaling is referred to as cis-signaling [2]. A soluble form of the IL6R (sIL6R) can be produced by processing of the receptor by proteases including disintegrin and metalloproteinase 17 (ADAM17) or by differential splicing [2] In contrast to other soluble receptors, the IL6-sIL6R complex act as an agonist and can induce signaling in cells which express gp130 and not IL6R. This kind of signal transduction is referred to as trans-signaling [2].

IL-6 plays an important role in promoting hepatic survival by stimulating liver regeneration, and by protecting the liver from damage caused by immune responses, alcohol and viral infection [3]. Despite its critical role in acute-phase response in the liver [4], IL6 signaling is protective during fibrosis progression [5], but promotes hepatocellular carcinoma (HCC) in response to chemical carcinogens [6] or in obese mice [7] and has been identified as a major factor associated with the sex disparity observed in liver cancer [6]. Serum IL6 levels are elevated in patients with chronic hepatitis B (CHB) and HCC [8,9,10] and perform better than IL-10, IL-12 and IFNα as a biomarker of clinical progression in HBV-related chronic liver diseases [11]. IL6 has been shown to suppress HBV replication and/or transcription in hepatoma cells [12], primary hepatocytes [13] and HBV transgenic mice [14].

Here we show that IL6 treatment leads to a reduction of cccDNA-bound histone acetylation paralleled by a rapid decrease in 3.5kb/pgRNA and subgenomic HBV RNAs transcription without affecting the cccDNA chromatinization or cccDNA levels. IL6 repressive effect on HBV replication is mediated by a loss of HNF1α and HNF4α binding to the cccDNA and a redistribution of STAT3 binding from the cccDNA to IL6 cellular target genes.

## Material and Methods

### Cell cultures, nucleic acid transfections and IL6 treatments

HepG2 hepatoma cells and the HepG2 derived clones HepG2.2.15 and NTCP-HepG2 [15] were cultured in Dulbecco’s modified Eagle’s medium (DMEM) supplemented with 10% fetal bovine serum (Gibco, Inc), 1% penicillin /streptomycin and 1% glutamine (Sigma) and maintained in a 5% CO2 humidified incubator at 37 C. Linear HBV monomers and siRNAs (HNF1α, HNF4α, STAT3 Smart-Pools from Dharmacon, Inc) were transfected into HepG2 human hepatoma cells using the Mirrus Bio trans IT-LT1 reagent (Mirrus) and the Lipofectamine Plus reagent (Invitrogen), respectively. rIL6 (Peprotech cat.no. 200–06) was used for 48 hours at a final concentration of 20ng/ml.

### Transient transfection of full-length HBV DNA genomes

Monomeric linear full-length wild-type (WT) HBV genotype A genomes were released from the pCR.HBV.A.EcoRI plasmid using EcoRI-PvuI (New England Biolabs) [16,17].

Briefly, HepG2 cells were seeded at a density of 2–3 million cells in 100-mm-diameter Petri dishes and transfected 24 hours later with 0.5 μg to 1 μg of digested HBV DNA. Unless specified otherwise, culture medium was changed 1 day after transfection and cells were harvested 48 hours post-transfection. All transfections included 0,1 μg of green fluorescence protein expression vector (GFP) to assess transfection efficiency (HepG2 cells range 28–32%). To exclude non-homologous recombination events at the level of the two ends of the transfected linear HBV DNA and the possible generation of circular HBV DNA molecules carrying sequence modifications at the recombination site, the HBV region spanning the predicted ends of the linear dsDNA was amplified and sequenced (data not shown). HBV cccDNA species in nuclear extracts from HepG2 transfected cells co-migrated in Southern blot analysis with the cccDNA isolated from HBV-infected NTCP-HepG2 cells, used as a positive control, and were converted into linear DNA by a single XhoI restriction digestion (data not shown).

### Virus production and NTCP-HepG2 cells infection

HBV virus was produced in HepG2.2.15 cells cultured in 2% DMSO for 15 days [18]. Cell culture medium was collected every three days and HBV particles were concentrated from clarified supernatants by overnight precipitation with 6% PEG 8000 (Sigma-Aldrich) and centrifugation at 4°C for 60 min at 10.000 rpm. The pellet was then resuspended in PBS1X with 10% FBS. 1/100 of viral stock was used for real time PCR quantification. NTCP-HepG2 cells infection was performed as previously described [15]. Briefly, cells were seeded in 6-well plates and inoculated overnight with approximately 6 × 10^2^ genome equivalents/cell in the presence of 4% polyethylene glycol 8000 and 2.5% DMSO (Sigma-Aldrich). rIL6 treatments (20 ng/ml for 48h) were carried out at day 10 post-infection.

### Core-particles (Cp) associated HBV DNA purification and quantitation

To purify HBV DNA from intracellular core particles, transfected cells were washed once with ice-cold phosphate buffered saline (PBS) and lysed in 10 mM Tris-HCl, pH 7.4, 1 mM EDTA, 50mM NaCl and 1% NP-40 (lysis buffer A). Nuclei were pelleted by centrifugation for 5 minute at 10,000 g. The supernatant was adjusted to 0.1 mM MgCl_2_ and treated with 0.1 mg/ml of DNase I for 30 minutes at 37°C. The reaction was stopped by adding EDTA to a final concentration of 1 mM. Viral core particles were precipitated in 0.8 M NaCl, 8% polyethylene glycol solution at 4°C for 1 hour. Core particles were then concentrated by centrifugation (10 minutes at 10.000 g) and were re-suspended in 10 mM Tris-HCl, 100 mM NaCl, 1 mM EDTA, 1% SDS and 0.5 mg/mL proteinase K and incubated for two hours at 56°C. Viral DNA released from lysed core particles was extracted with phenol-chloroform (1:1), precipitated with isopropanol and quantified by real time PCR in a Light Cycler instrument (Roche, Inc). PCR reactions were performed in 20 μL volume containing 3 mM MgCl_2_, 0.5 μM forward and reverse primers, 0.2 μM of 3’-fluorescein (FL)-labeled probe, and 0.2 μM of 5’-Red640 (R640)-labeled probe. The following HBV primers and probes were used: forward 5’-CTCGTGGTGGACTTCTCTC-3’ (nt 256–274), reverse 5’-CAGCAGGATGAAGAGGAA-3’ (nt 421–404) and the specific FRET hybridization probes: 5’-CACTCACCAACCTCCTGTCCTCCAA-FL-3’, Red640-5’-TGTCCTGGTTATCGCTGGATGTGTCT-3’. Amplifications were performed as follows: 95°C for 5 minutes followed by 45 cycles at 95°C for 10 seconds, 57°C for 10 seconds and 72°C for 20 seconds. The β-globin housekeeping gene was used to normalize the DNA samples [primers and probes: forward 5’-ACACAACTGTGTTCACTAGC-3’, reverse 5’-CAACTTCATCCACGTTCACC-3’ and the specific FRET hybridization probes 5’-CAAACAGACACCATGGTGCACCTGACTCCTGAGGA-FL-3’, Red640-5’-AAGTCTGCCGTTACTGCCCTGTGGGGCAA-3’. HBV-DNA complete genomes from Clonit Srl (cat.no. 05960116) were used for the standard curve.

### HBV cccDNA quantification

HepG2 cells were collected at the indicated times after transfection, re-suspended in lysis buffer A (see above) and incubated 10 minutes at 4°C. Lysates were centrifuged 5 minute at 10.000 g, pelleted nuclei resuspended in lysis buffer B (10 mM Tris-HCL, 10 mM EDTA, 150 mM NaCl, 0,5% SDS and 0.5 mg/ml proteinase K) and incubated overnight at 37°C after 1 or 2 pulses of sonication at 80% power. Nucleic acids were then extracted by phenol-chloroform (1:1) and ethanol precipitated. 500 ng aliquots of each extracted DNA were treated for 45 minutes at 37°C with 10 U of Plasmid safe DNAseI (Epicentre Inc.). DNAse was inactivated by incubating the reactions for 30 minutes at 70°C. Real time PCR experiments were performed in a Light Cycler using a 20 μL reaction volume containing 100 ng of DNA, 3 mM MgCl_2_, 0.5 μM forward and reverse primers, 0.2 μM of 3’-fluorescein (FL)-labeled probe, and 0.2 μM of 5’-Red640 (R640)-labeled probe. Forward and reverse primers were: 5’-CTCCCCGTCTGTGCCTTCT-3’ (nt 1548–1566) and 5’-GCCCCAAAGCCACCCAAG-3’ (nt 1903–1886), respectively, whereas the hybridization probes were 5’-GTTCACGGTGGTCTCCATGCAACGT-FL-3’ and 5’-R640-AGGTGAAGCGAAGTGCACACGGACC-3’, respectively. Amplification was performed as follows: 95°C for 10 minutes then 45 cycles of 95°C for 10 seconds, 58°C for 6 seconds, 63°C for 10 seconds and 72°C for 20 seconds. The β-globin housekeeping gene was used to normalize the DNA samples, using the primers and probes described above. HBV DNA complete genomes from Clonit Srl (cat.no.05960116) were used to build the standard curves.

### HBV RNAs and cellular mRNA analysis

Total RNA was extracted using the TRIzol reagent (Invitrogen) as recommended by the manufacturer. The RNA samples were treated with RQ1 RNase-Free DNase (Promega Inc.) for 60 minutes at 37°C and stored until used. RNA quality and quantity were monitored by ethidium-bromide staining and by UV absorbance. For viral RNAs analysis, 2 μg of DNase-treated RNA was reverse transcribed and amplified by the ThermoScript RT-PCR System (Invitrogen). Then 2 μl of each cDNA was quantified by real-time PCR analysis. The same primers and probes designed for core particles associated HBV DNA quantification were used to evaluate total HBV RNA levels (corresponding to S [2.1 Kb], pre-S [2.4 Kb], and 3.5Kb-pregenome mRNAs). To quantitate the 3.5Kb/pgRNA species we used the following selective primers and probes that do not detect the S [2.1 Kb], pre-S [2.4 Kb] or HBx [0.7 Kb] HBV RNAs: forward primer, 5′-GCCTTAGAGTCTC CTGAGCA-3′, (nt 2019–2048); reverse primer, 5′-GAGGGAGTTCTTCTTCT AGG-3′ (nt 2380–2399); FRET hybridization probes, 5′–AGTGTGGATTCGCA CTCCTCCAGC-FL-3′, and Red640-5′ATAGACCACCAAATGCCCCTATCTTAT CAAC-3′. The amount of pre-S/S RNA was estimated by subtracting the 3.5Kb/pgRNA quantity from the total HBV RNA amount [19]. The h-G6PDH house-keeping gene Light Cycler set (Roche Diagnostics) was used to normalize the RNA samples.

For Northern blot analysis, 5 μg of total RNA per sample was separated on a 1% formaldehyde-agarose gel and blotted onto Zeta-Probe GT membranes (Bio-Rad Laboratories). Radioactive probes were prepared by random priming protocol, using either full-length HBV DNA and 32P α-dCTP (Amersham). After hybridization, the membrane was washed and exposed to X-Omat film (Kodak Inc.) at –80°C. 28S/18S rRNAs were used as an internal control for sample loading. Densitometric analysis of the 3.5Kb/pgRNA bands was performed using the open source ImageJ software.

Cellular transcripts were quantified by SYBR Green real time PCR (Roche Applied Science) using the following primers: HNF1α forward 5’-AGCGAGAGACGCTAGTGGAG-3’, reverse 5’-CCGGAA GGCTTCTTCTTTG-3’, HNF4α forward 5’-CACTCAACGAGAACCAGCAG-3’, reverse 5’-TGTCCC GACAGATCACCTC-3’, HP forward 5’-TGAATGTGAAGCAGTATGTGGGA-3’, reverse 5’-CATTGATCAGCGTGGCACCT-3’. β-actin amplification was used to normalize the RNA samples [primers: forward 5’-GCACTCTTCCAGCCTTCCT-3’, reverse 5’-AGGTCTTTGCGGATGTCCAC-3’].

### Immunoblotting

To prepare nuclear extracts HepG2 cells were lysed with hypotonic buffer, pellet were resuspended with 500 μl of PIPES buffer [5 mM PIPES (piperazine N,N bis zethone sulfonic acid) pH 8, 85 mM KCl, 0,5% NP40] plus protease inhibitor cocktail (PIC, Sigma p8340) and left 10 min on ice. The nuclei were separated by centrifuge at 10.000 g for 5 minute a 4°C. Nuclei were lysed with RIPA buffer (50 mM Tris-HCL ph8, 150 mM NaCl, 1% NP40, 0,5% sodium deoxycolate, 0.1% SDS). Total protein extracts were obtained by lysing the cells directly the with RIPA buffer. Protein concentrations were determined using the Bradford Protein Assay Reagent (Biorad). Protein lysates were separated by SDS-PAGE gels, transferred to nitrocellulose membranes and incubated with the following antibodies: anti-HNF1α (Santa Cruz Biotechnology, #sc6547), anti-HNF4α (Santa Cruz Biotechnology, #sc6556), anti-STAT3 (Cell signaling, #9139), anti-phospho-STAT3 (Cell signaling #9131), laminB (Santa Cruz Biotechnology, #sc6216) and β-actin (Santa Cruz Biotechnology, #sc1616).

### ChIP assays

48 hours after transfection with linear HBV monomers, HepG2 cells were resuspended in 1 ml of PIPES buffer plus PIC and incubated 10 minutes at 4°C. Lysates were centrifuged at 10,000 g for 5 minutes to pellet the nuclei. The supernatant was removed, and the nuclei fixed in 1% formaldehyde for 30 minutes at 4°C. Isolated cross-linked nuclei were incubated with SDS lysis buffer (1% SDS, 10 mM EDTA, 50 mM Tris-chloride pH 8.1), plus PIC, for 30 minutes on ice. The resulting chromatin solution was sonicated for 10 pulses of 45 seconds at 80% power to generate 300-1000-bp DNA fragments using a BioRuptor Sonicator (Diagenode Inc). After microcentrifugation, the supernatant was diluted 1:10 in a buffer 0.01% SDS, 1.1% Triton X-100, 1.2 mM EDTA, 16.7 mM Tris-chloride, pH 8.1, 167 mM NaCl buffer containing protease inhibitors, pre-cleared with blocked Protein G Plus (Pierce), and divided into aliquots. The chromatin was then subjected to immunoprecipitation for 14–16 hours at 4°C using antibodies specific to H3 (Abcam #ab1791), AcH3 (Upstate, #07–352), HDAC1 (Upstate, #06–720); STAT3 (Cell signaling, #9139) and phospho-STAT3 (Cell signaling, #9131). Immunoprecipitations with non specific immunoglobulins (Abcam #27478) were included in each experiment as a negative control. After the reverse cross-linking, immunoprecipitated chromatin was purified by phenol/chloroform (1:1) extraction and ethanol precipitation and analyzed by real-time PCR amplification using primers and probes specific for the HBV cccDNA (see above). Lack of amplification of immunoprecipitated chromatin with primers spanning the predicted ends of the transfected linear dsDNA confirmed the specificity of the ChIP assay for the cccDNA-like circular molecules vs the transfected linear dsHBV-DNA (data not shown). For the amplification of Haptoglobin (HP) promoter containing STAT3 binding sites we use the following primers: forward 5’-ACTGGTACCCACAAGAAAATCAAG TGTGAAGCA-3’ and reverse 5’-GTTGG TCTTGCCTCTGGAAGAGCAGTG-3’.

### Statistics

P values were determined using the 2-tailed Student’s t test. *0,01≤ P< 0,05; **0,001≤ P< 0.01; *** P < 0,001.

## Results and Discussion

### IL6 targets the epigenetic regulation of cccDNA minichromosome

**Fig 1:**
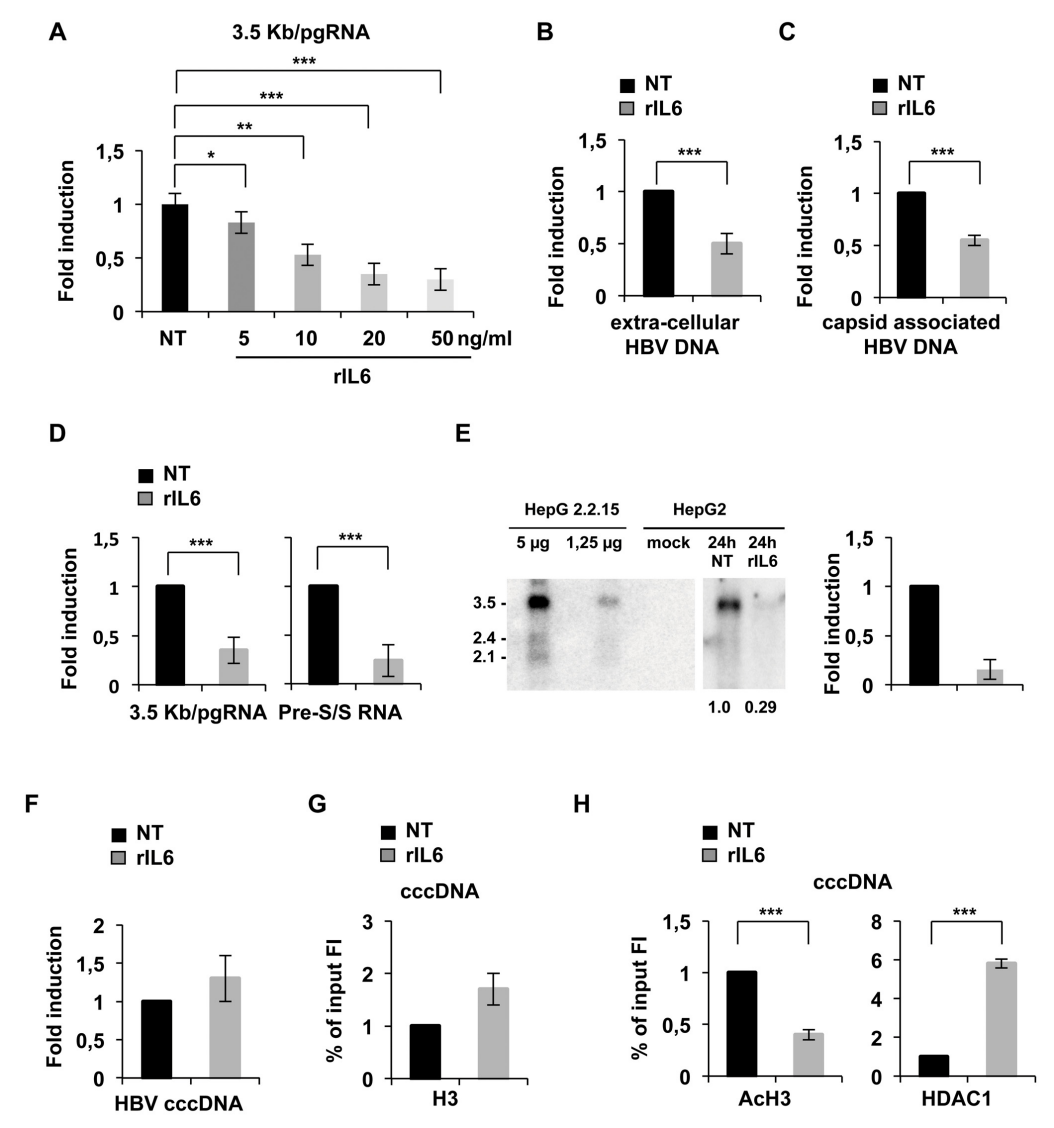
IL-6 inhibits HBV replication and cccDNA transcription in HepG2 cells transfected with HBV monomers. HepG2 cells were transfected with monomeric linear full length wild type HBV *adw* (genotype A) genomes and were harvested after 48 hours. **A)** HepG2 transfected cells were left untreated or exposed to 5, 10, 20, 50 ng/ml of rIL6. The 3.5Kb/pgRNA species were quantified using selective primers and probes that do not detect the S [2.1 Kb], pre-S [2.4 Kb] or HBx [0.7 Kb] HBV RNAs. **B)** HBV DNA was extracted from viral particles isolated from the medium of untreated and rIL6 treated HepG2 transfected cells and quantified by real time qPCR using primers annealing to the S region. **C)**. Cytoplasmic HBV core particles were isolated from untreated and IL6 treated transfected cells and total core particles associated HBV DNA was quantified as in B). The β-globin housekeeping gene was used to normalize the DNA samples. **D)** RNAs were isolated from untreated and IL6-treated HepG2 transfected cells. The 3.5Kb/pgRNA species were quantified as described in A). To evaluate total HBV RNA levels (corresponding to S [2.1 Kb], pre-S [2.4 Kb], and 3.5Kb-pregenome mRNAs) we used primers and probes that anneal to the S region and detect the S [2.1 Kb], pre-S [2.4 Kb] and 3.5Kb-pregenome but not the HBx [0.7 Kb] HBV RNAs. The amount of pre-S/S RNA was estimated by subtracting the 3.5Kb/pgRNA quantity from the total HBV RNA amount. hG6PDH mRNA amplification was used to normalize for equal loading of each RNA sample. All results in **A-D)** are expressed as arbitrary units and the histograms show the mean from three independent experiments; bars indicate S.D. **E)** Northern blot analysis of untreated and IL6-treated HepG2 transfected cells (48 hours; 20 ng/ml). 28S/18S rRNAs were used as an internal control for sample loading. 5 and 1.25 μg of RNAs extracted from the HepG2-derived 2.2.15 cell line were used as HBV positive loading controls. Figures represent the relative intensity of the 3.5Kb/pgRNA band normalized to the 28S rRNA. *Right Panel*: Densitometric quantification of HBV 3.5Kb/pgRNA was performed using the open source ImageJ software. **F)** cccDNA was extracted from the nuclei of untreated and IL6-treated HepG2 transfected cells. qPCR analysis was performed using cccDNA selective primers and β-globin primers to normalize the DNA samples. Results are expressed as in A-D). **F-H)** Cross-linked chromatin is extracted from HepG2 cells transfected with monomeric linear full-length HBV DNA and treated for 48 hours with rIL6. The cross-linked chromatin was immunoprecipitated with a relevant control IgG or specific anti-H3 (Fig 1G), anti-AcH3 (Fig 1H, left panel), anti-HDAC1 (Fig 1H, right panel) antibodies. Immunoprecipitated chromatin samples were analyzed by real time PCR with HBV cccDNA selective primers. ChIP results are expressed as Fold Induction (FI) of the % of Input and the histograms show the mean from three independent experiments; bars indicate S.D. * 0,01 ≤ P < 0,05; ** 0,001 ≤ P < 0.01; *** P < 0,001.

In order to investigate whether IL6 signaling may exert a direct effect on cccDNA transcription we first made use of an established cccDNA-driven HBV replication model [17,18,20,21]. HepG2 cells were transfected with WT genotype A HBV DNA linear monomers in the presence or absence of recombinant IL6 (rIL6, 20 ng/ml). 48 hours post-transfection cells and cell culture supernatants were harvested and processed in parallel to extract extracellular total HBV DNA, cytoplasmic core particles-associated HBV-DNA, total RNA and nuclear DNA [17]. Total HBV-DNA, HBV-RNAs and cccDNA levels were quantified by qPCR as previously described [17,19]. IL6 treatments were carried out at 20 ng/ml according to the dose response curve shown in Fig 1A. Exposure to rIL6 significantly reduces the levels of extracellular HBV-DNA (Fig 1B), capsid-associated HBV DNA (Fig 1C), 3.5Kb/pgRNA (Fig 1D, left panel) and pre-S/S RNAs (Fig 1D, right panel). Northern blot experiments confirmed the strong reduction of the 3.5Kb/pgRNA species (Fig 1E), that are the predominant HBV transcripts in HepG2 cells and all HepG2 derivative clones. Importantly, IL6 does not reduce cccDNA levels (Fig 1F), thus suggesting a direct transcriptional effect underlying the observed reduction of viral RNA steady state levels.

Increasing evidence support the notion that cccDNA transcription is controlled by cccDNA-bound histones post-translational modifications, that results from the coordinated binding onto the viral mini-chromosome and the activity of the viral proteins HBx and HBc [17,21,22], cellular transcription factors [23,24] and chromatin modifying enzymes [17,21,23,24,25,26,27]. We used the cccDNA ChIP assay [17,20,21], that couples a chromatin immuno-precipitation step with a cccDNA-specific real time PCR to selectively detect histones and non-histone proteins bound to the cccDNA. We found that IL6 treatment does not reduce but rather slightly increases the level of H3 histone-bound cccDNA (Fig 1G) whereas cccDNA-bound histones are significantly hypo-acetylated (Fig 1H, left panel) and there is a strong recruitment of the histone de-acetylase HDAC1 (Fig 1H, right panel). Although additional mechanisms, such as a reduced mRNA stability, may in principle contribute to the reduction of steady state HBV transcripts levels in rIL6-treated cells, the observed changes in the cccDNA chromatin and the recruitment of histone deacetylases on the minichromosome strongly implicate transcriptional events.

**Fig 2:**
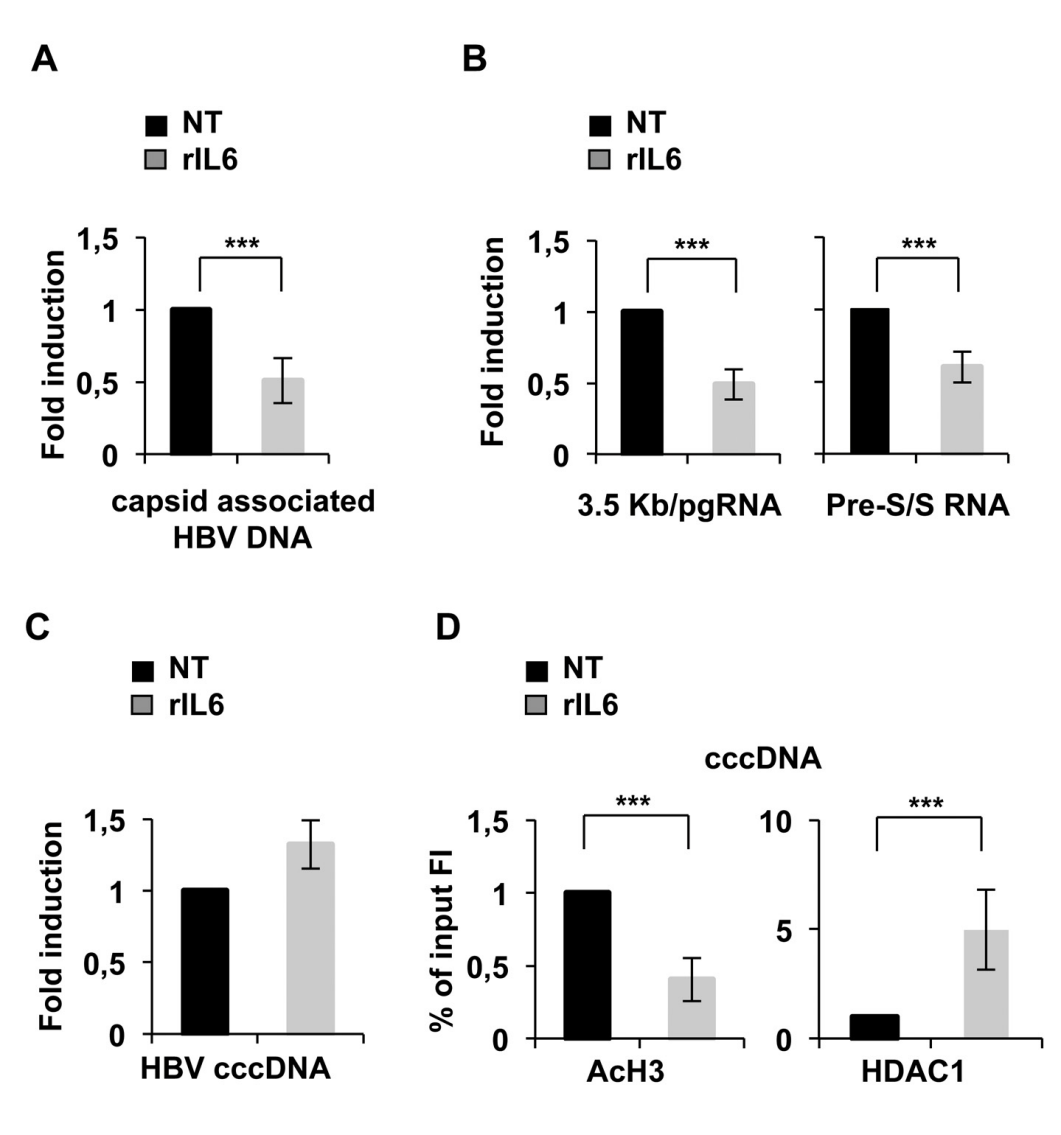
IL-6 inhibits cccDNA transcription activity in HBV infected NTCP-HepG2 cells. HepG2-NTCP cells were infected with 6 × 10^2^ genome equivalents/cells of HBV in and treated with rIL6 for 48 hours at day 10 post-infection. **A)** Cytoplasmic HBV core particles were isolated from untreated and IL6 treated infected cells and total core particles associated HBV DNA was quantified as described in the Legend to Fig 1C). **B)** RNAs were isolated from untreated and IL6-treated HepG2-NTCP infected cells. The 3.5Kb/pgRNA and the pre-S/S RNA were quantified and described in the legend to Fig 1D. **C)** cccDNA was extracted from the nuclei of untreated and IL6-treated infected cells. qPCR analysis was performed using cccDNA selective primers and β-globin primers to normalize the DNA samples. All results in **A-C)** are expressed as arbitrary units and the histograms show the mean from three independent experiments; bars indicate S.D. **D)** Cross-linked chromatin is extracted from the nuclei of NTCP-HepG2 cells treated or not with with rIL6 for 48 hours at day 10 post-infection. The cross-linked chromatin was immunoprecipitated with a relevant control IgG or specific anti-AcH3 and anti-HDAC1 antibodies. ChIPs were analyzed and the results expressed as described in the Legend to Fig 1F. * 0,01 ≤ P < 0,05; ** 0,001 ≤ P < 0.01; *** P < 0,001.

These results were fully confirmed in HBV infected NTCP-HepG2 cells treated for 48 hours with rIL6 after 10 days of infection, when a stable pool of cccDNA is established (see [15] and Belloni unpublished observations]. As shown in Fig 2A, IL6 treatment significantly reduces HBV replication and cccDNA transcription (Fig 2B) without affecting cccDNA levels (Fig 2C). The reduction in the steady state levels of 3.5Kb/pgRNA and HBV 2.4Kb and 2.1Kb sub-genomic transcripts in response to IL-6 is paralleled by a strong reduction of cccDNA-bound H3 histone acetylation (Fig 2D, left panel) and an increase of HDAC1 recruitment onto the HBV minichromosome (Fig 2D, right panel).

Altogether, these results indicate that IL6 repression of HBV replication is not mediated by the inhibition of cccDNA formation and/or its chromatinization but rather by a direct impact on the epigenetic control of cccDNA transcription.

### IL6 reduces P-STAT3, HNF1α and HNF4α binding on the cccDNA

**Fig 3:**
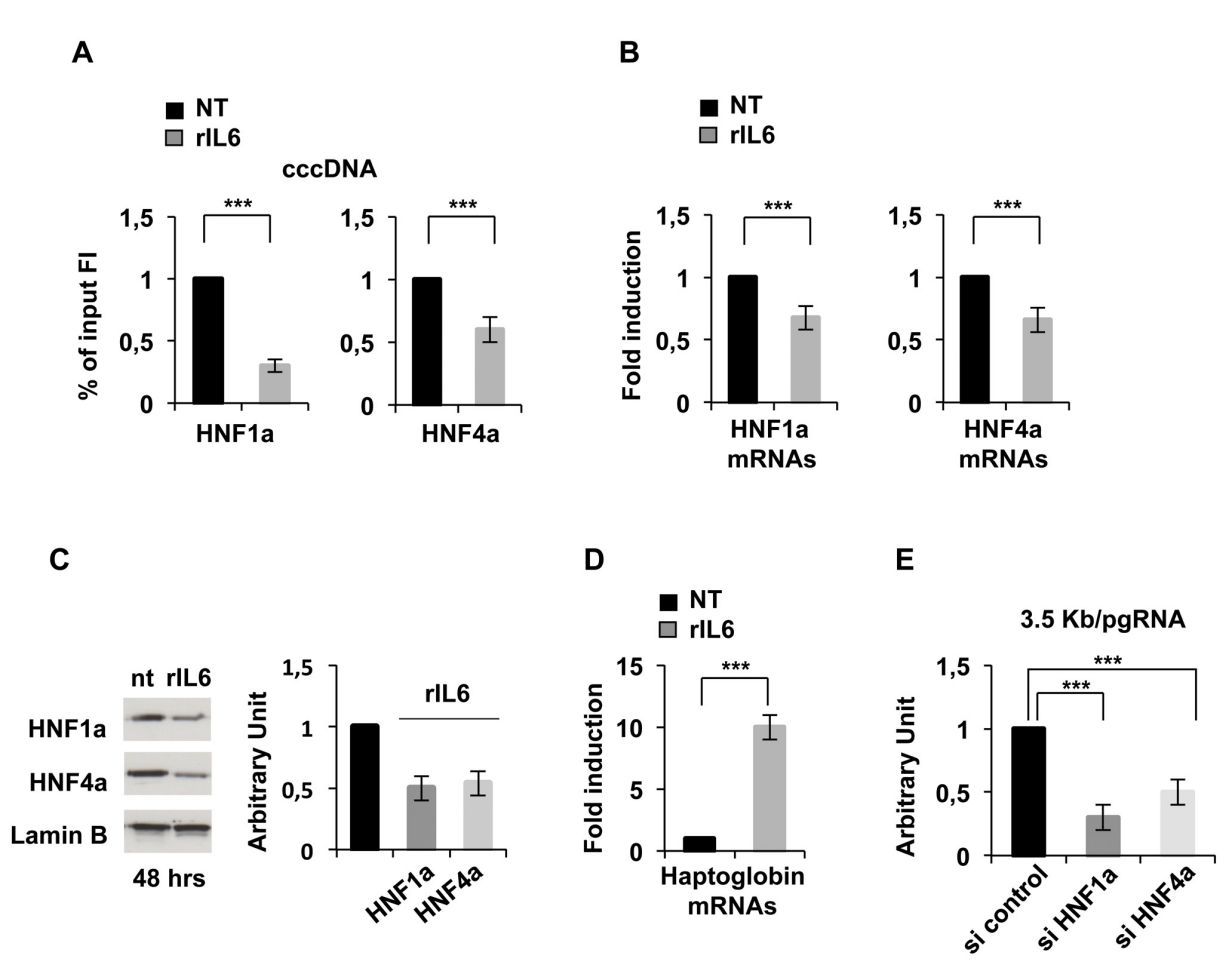
IL6 decreases HNF1α and HNF4α occupancy on the cccDNA. **A)** Anti-HNF1α and anti-HNF4α cccDNA ChIP assay. HepG2 transfected cells were treated with rIL6 for 48 hours. Immunoprecipitated chromatin samples were analyzed by real time qPCR with HBV cccDNA selective primers (see Legend to Fig 1). ChIP results are expressed as Fold Induction (FI) of the % of Input and the histograms show the mean from three independent experiments; bars indicate S.D. **B)** Cellular RNAs were extracted from untreated and IL6 treated HepG2 cells. HNF1α and HNF4α were quantified by SYBR Green real time PCR. **C)** Nuclear extracts were analyzed by western blot. 30μg of nuclear proteins were analyzed for HNF1α, HNF4α, and β-lamin as control. **D)** Haptoglobin gene expression was measured by SYBR Green real time PCR. β-actin amplification was used in B) and D) to normalize the RNA samples. **E)** HepG2 cells were transfected with control or HNF1α or HNF4α siRNA pools. After 48 hours pgRNA was extracted and quantified as in Fig 1D.

Several binding sites for ubiquitous and liver-specific transcription factors have been described within the HBV promoters and enhancer I/II regions [28]. Among these we focused on the hepatocyte nuclear factor (HNF) 1α and HNF 4α, two transcription factors known to be essential for HBV replication [29,30], and on STAT3/Phospho-STAT3, the major transcriptional effector of IL6 signaling [1]. Using anti-HNF1α and anti-HNF4α cccDNA ChIP assays we found that HNF1α and HNF4α bind to the cccDNA in HBV replicating cells and that their recruitment is strongly reduced after 48 hours of IL6 treatment (Fig 3A). IL6, produced by Kuppfer cells exposed to HBV infection, has been shown to down-regulate HNF1α and HNF4α levels in hepatocytes [13]. Similarly, a loss of HNF4α has been also involved in TGFβ1 suppression of HBV replication [31]. We confirmed the reduction of both HNF1α and HNF4α transcripts (Fig 3B) and protein levels (Fig 3C) in response to IL6 treatment in our cells. As expected, the mRNA levels of Haptoglobin (HP), a known IL6/STAT3 target gene [32], are strongly increased in response to IL6 (Fig 3D). The role of HNF1α and HNF4α in 3.5Kb/pgRNA transcription from the cccDNA is underlined by the significant impact on 3.5Kb/pgRNA levels of the siRNA mediated reduction of HNF1α and HNF4α expression (HNF1α and HNF4α Smart-Pools, Dharmacon, Inc.) (Fig 3E).

Altogether, these results indicate that the reduced recruitment of HNF1α and HNF4α on the cccDNA in IL-6 treated cells reflects the negative effect of IL6 on HNF1α and HNF4α levels and contributes to IL6-mediated inhibition of HBV replication. Activation of JNK and, to a lesser extent, ERKs mediates IL6 down-regulation of HNF1α and HNF4α in primary hepatocytes [13] but the precise mechanism is not known. Recent evidence links IL6/STAT3-mediated activation of miR-24 and miR-629 with HNF4α silencing and HCC development [33]. In silico analisys of putative miRNA targets identifies miR-24 and miR-33a, that are induced by IL-6 in liver cells, as potential effectors of IL6-mediated repression of both HNF1α and HNF4α [34] but further experiments are needed to establish a role for STAT3 regulated miRNAs and IL-6 antiviral effects on HBV.

**Fig 4:**
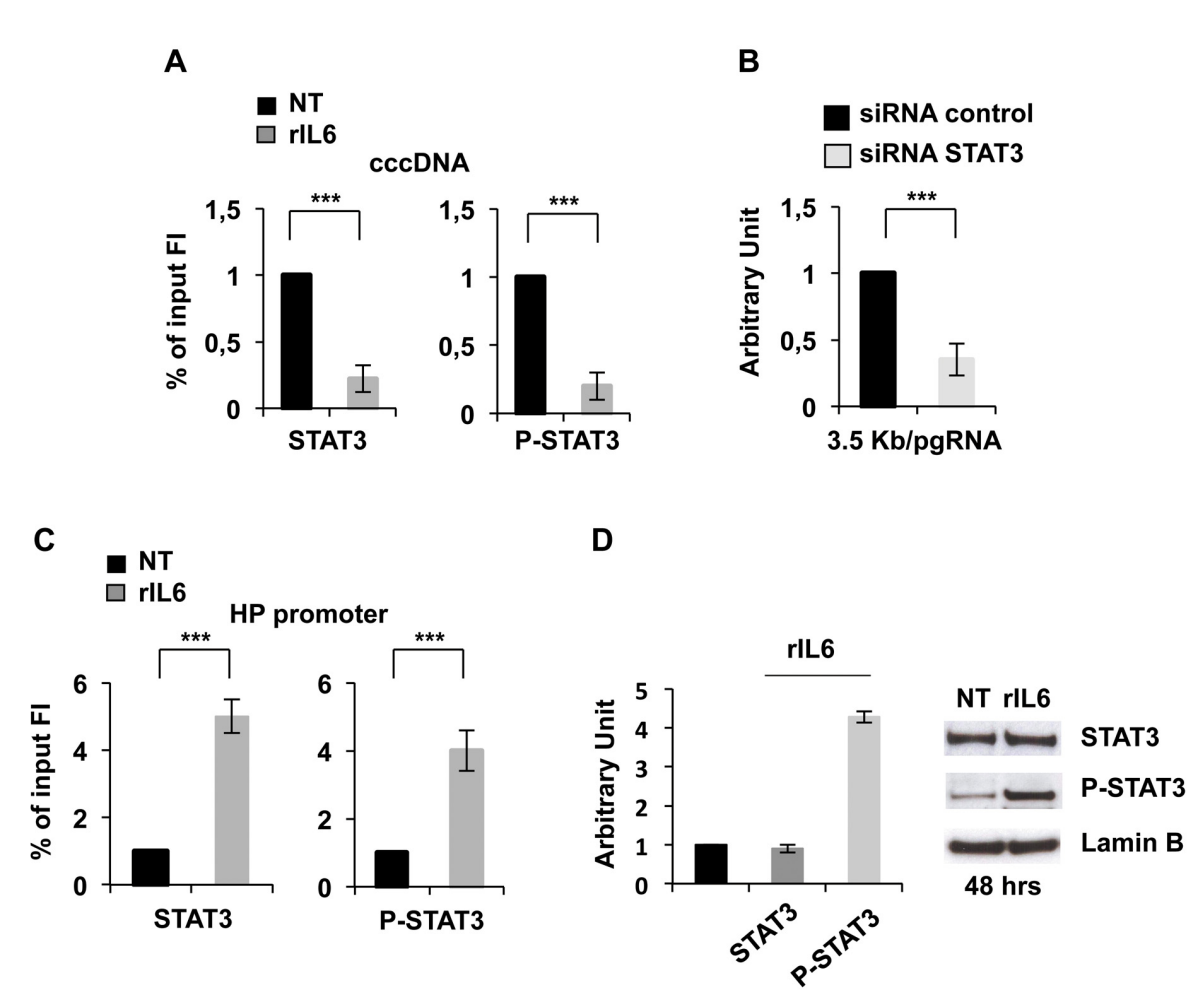
Modulation of P-STAT3 chromatin binding by IL6. In upper panel, **A)** Anti-STAT3 and anti-P-STAT3 chromatin immuno-precipitations were performed as in Fig 2A and analyzed with cccDNA specific primers (see Legend to Fig 1). **B)** HepG2 cells were transfected with monomeric linear full-length HBV DNA in combination with the indicated siRNA pools. After 48 hours, total RNA was extracted and pgRNA levels analyzed by qPCR as described in Legend to Fig 1D. **C)** Anti-STAT3 and anti-P-STAT3 immuno-precipitates were analyzed with primers specific for the Haptoglobin (HP) promoter. All ChIP results are expressed as Fold Induction (FI) of the % of Input and the histograms show the mean from three independent experiments; bars indicate S.D. **D)** 30 μg of nuclear proteins were analyzed by immunoblot with anti-STAT3, anti-P-STAT3 and anti-lamin B (loading control) antibodies (*left panel*). Densitometric quantitation of STAT3, P-STAT3 and lamin B immunoblots are shown in the *right panel*.

Although IL-6 has a negative effect on HBV replication [12,13] some observations indicate that STAT3 might also have opposite effects [35, 36] and contribute to HBV reactivation in response to radiation therapy [37]. To better investigate the role of STAT3/P-STAT3 in the control of cccDNA transcription and in IL-6 induced repression of cccDNA transcription we performed additional anti-P-STAT3 cccDNA ChIP experiments in HepG2 replicating cells. As shown in Fig 4A, both total and phosphorylated STAT3 are actively recruited onto the cccDNA when HBV replication is high, whereas their binding to the cccDNA is dramatically reduced after IL6 treatment (Fig 4A). Notably, siRNA mediated silencing of STAT3 expression (STAT3 Smart Pool, Dharmacon, Inc) decreases 3.5Kb/pgRNA RNA transcription, thus linking STAT3/P-STAT3 occupancy with cccDNA transcription ad HBV replication (Fig 4B). Notably, in contrast to what we observed on the cccDNA, the anti-P-STAT3 ChIP assay showed a sharp increase of STAT3 and P-STAT3 occupancy on the IL-6 Responsive Element (IL-6 RE) of the Haptoglobin promoter (Fig 4C). Differently from what was observed for HNF1α and HNF4α, the reduction of P-STAT3 binding to the cccDNA is not explained by a reduction of STAT3 protein √nthlevels, that are unaffected by IL-6, or P-STAT3 that, as expected, increases after IL6 treatment (Fig 4D). Thus, the loss of P-STAT3 binding on the cccDNA is not due to a generalized negative regulation of P-STAT3 ability to bind its genomic targets but rather reflects specific changes imposed by STAT3 signaling on the cccDNA chromatin environment in HBV replicating cells or a relative higher affinity to genomic target sites vs the STAT3 binding site on the cccDNA.

**Fig 5:**
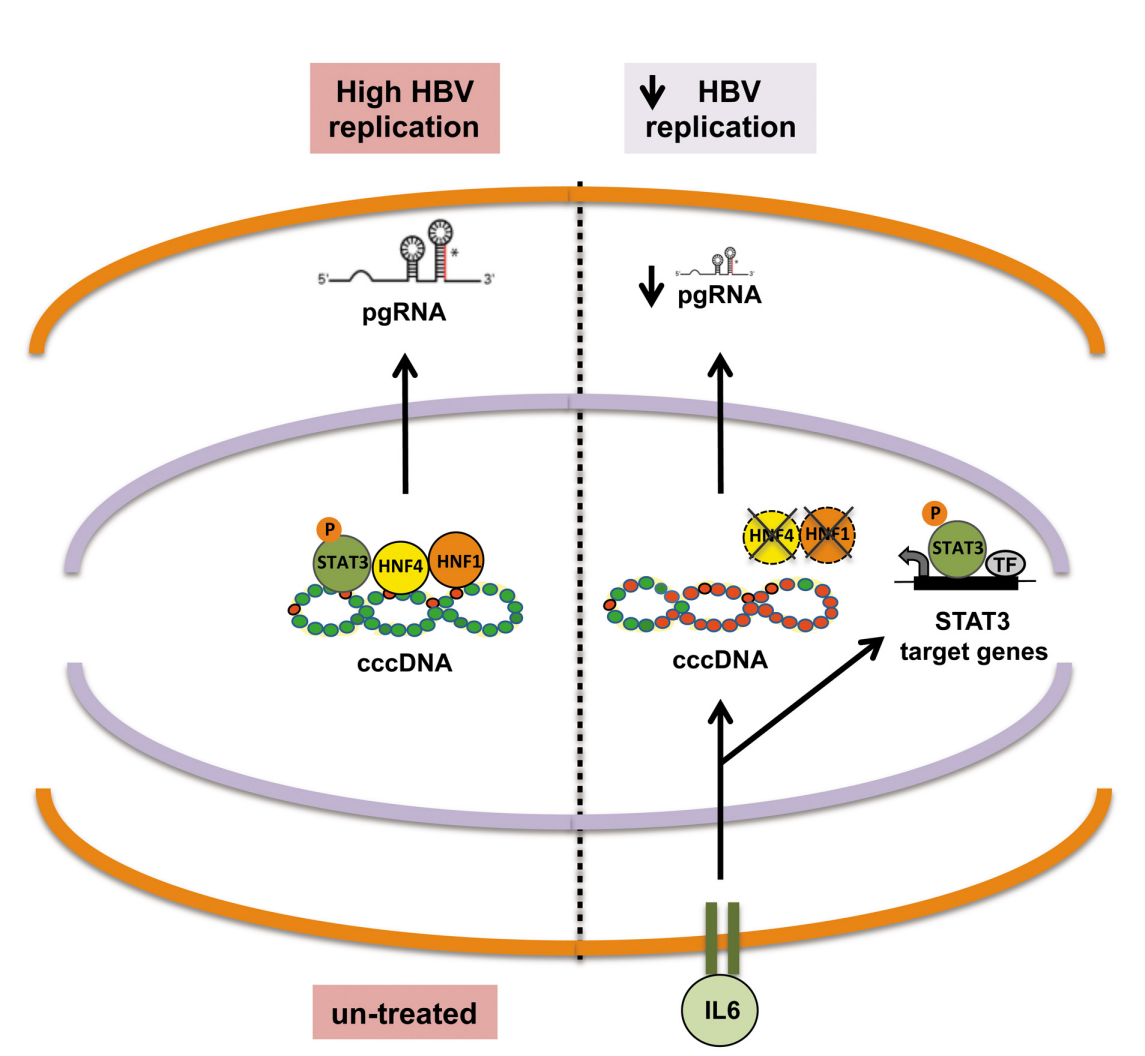
Schematic model of IL6 modulation of cccDNA transcription. STAT3, HNF1α and HNF4α bind the cccDNA and contribute to activate the transcription of cccDNA (middle). IL6 treatment results in the hypo-acetylation of cccDNA-bound histones and inhibits HBV transcription through the combined effect on HNF1α and HNF4α protein levels and a lower recruitment of P-STAT3 to the cccDNA, as compared to cellular promoters (i.e. Haptoglobin).

In conclusion, our results support a model to explain the antiviral activity of IL6 towards HBV (Fig 5) where IL6 inhibits cccDNA transcription by reducing the binding of essential transcriptional factors (HNF1α, HNF4α and STAT3) onto the cccDNA and leading to cccDNA-bound histones hypo-acetylation and cccDNA silencing. Notably, the mechanisms by which IL6 affects cccDNA transcription and HBV replication are different from those reported for IFNα that induces the hypoacetylation of cccDNA-bound histones [20, 38] and the recruitment of the PRC2 repressive complex [20].
