# Correction: IL6 Inhibits HBV Transcription by Targeting the Epigenetic Control of the Nuclear cccDNA Minichromosome

**DOI:** 10.1371/journal.pone.0145555

**Published:** 2015-12-16

**Authors:** Gianna Aurora Palumbo, Cecilia Scisciani, Natalia Pediconi, Leonardo Lupacchini, Dulce Alfaiate, Francesca Guerrieri, Ludovica Calvo, Debora Salerno, Silvia Di Cocco, Massimo Levrero, Laura Belloni

The fifth author’s name is spelled incorrectly. The correct name is: Dulce Alfaiate. The correct citation is: Palumbo GA, Scisciani C, Pediconi N, Lupacchini L, Alfaiate D, Guerrieri F, et al. (2015) IL6 Inhibits HBV Transcription by Targeting the Epigenetic Control of the Nuclear cccDNA Minichromosome. PLoS ONE 10(11): e0142599. doi:10.1371/journal.pone.0142599
